# Mitochondria, Estrogen and Female Brain Aging

**DOI:** 10.3389/fnagi.2018.00124

**Published:** 2018-04-27

**Authors:** Imane Lejri, Amandine Grimm, Anne Eckert

**Affiliations:** ^1^Neurobiology Lab for Brain Aging and Mental Health, Transfaculty Research Platform Molecular and Cognitive Neuroscience, University of Basel, Basel, Switzerland; ^2^Psychiatric University Clinics, University of Basel, Basel, Switzerland

**Keywords:** neurodegeneration, female brain aging, mitochondria, estrogen, protection, bioenergetics, SIRT3, BDNF

## Abstract

Mitochondria play an essential role in the generation of steroid hormones including the female sex hormones. These hormones are, in turn, able to modulate mitochondrial activities. Mitochondria possess crucial roles in cell maintenance, survival and well-being, because they are the main source of energy as well as of reactive oxygen species (ROS) within the cell. The impairment of these important organelles is one of the central features of aging. In women’s health, estrogen plays an important role during adulthood not only in the estrous cycle, but also in the brain via neuroprotective, neurotrophic and antioxidant modes of action. The hypestrogenic state in the peri- as well as in the prolonged postmenopause might increase the vulnerability of elderly women to brain degeneration and age-related pathologies. However, the underlying mechanisms that affect these processes are not well elucidated. Understanding the relationship between estrogen and mitochondria might therefore provide better insights into the female aging process. Thus, in this review, we first describe mitochondrial dysfunction in the aging brain. Second, we discuss the estrogen-dependent actions on the mitochondrial activity, including recent evidence of the estrogen—brain-derived neurotrophic factor and estrogen—sirtuin 3 (SIRT3) pathways, as well as their potential implications during female aging.

## Introduction

Mitochondria play crucial roles in different aspects of cellular physiology, including calcium homeostasis, metabolism, apoptosis and ATP production (Rizzuto et al., 2012). At the electron transport chain (ETC), especially at the respiratory complexes I and III, mitochondria produce reactive oxygen species (ROS; Quinlan et al., 2013). Mitochondrial respiration via ETC generates a flux of electrons that is coupled to a gradient of protons. During the oxidative phosphorylation (OXPHOS), electrons are neutralized to water upon reaction with oxygen at complex IV (cytochrome *c* oxidase (COX)), while the gradient of protons is necessary for the ATP synthesis via complex V (ATP synthase; Velarde, 2014).

**Figure 1:**
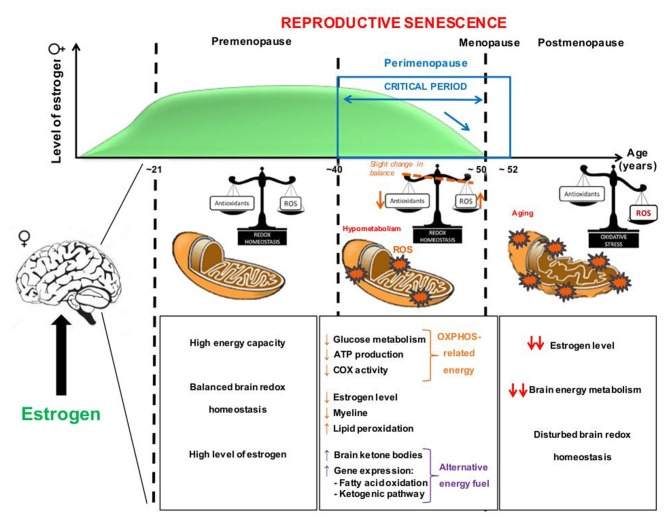
Potential sequence of pathological events occurring at the mitochondrial level during aging and «the critical time period» of decline in estrogen (E2) in female brain. During the premenopause, high levels of estrogen are paralleled by a normal mitochondrial activity with a balanced redox homeostasis and a high brain energy metabolism. At the beginning of the reproductive senescence, or perimenopause, a decrease of estrogen levels is accompanied with an increase of oxidative stress (ROS levels) and, consequently, hypometabolism. During aging, alterations of the mitochondrial membrane lipid profile are also reported. Cells possess compensatory mechanisms, as antioxidant defenses, to keep the system in balance. In case the compensatory system is exhausted, metabolic impairments may occur, such as a decrease of ATP levels, glucose hypometabolism and mitochondrial respiration (e.g., cytochrome c oxidase (COX) activity) as well as myelin impairments. In parallel, an increased expression of genes involved in fatty acid oxidation and the ketogenic pathway were reported as an alternative fuel for cells. The “critical period” (represented by the blue arrow) indicates a fast drop of estrogen levels in a relatively short time period, suggesting a period of women’s life where they are more vulnerable and more likely to develop age-related brain disorders such as Alzheimer’s disease (AD). Estrogen replacement therapies (ERT) may be beneficial before women reach a critical threshold of cellular damage during perimenopause and early after menopause. ROS, reactive oxygen species.

Mitochondria are also essential sites for steroid hormone biosynthesis including estrogen, the main female steroid hormone (Vest and Pike, 2013). Estrogen is involved in many physiological functions such as modulation of the effects of the trophic factors in the brain, enhancement of the cerebral blood flow, and prevention of atrophy of cholinergic neurons (Castellani et al., 2010). The estrogen family (E2) includes the hormones estrone, estradiol (17β-estradiol) and estriol, all of them playing a role in the estrous cycle. Regarding to the estrogenic effect, estradiol is the predominant form of estrogen and ten times as potent as estrone and about 80 times as potent as estriol. During reproductive senescence, a hormonal deficit occurs in menopausal women, characterized by a sudden decline in circulating estrogen level (Figure 1; Vest and Pike, 2013). Menopause officially marks the end of female reproduction. Postmenopause represents the years after the menopause while the transitional stage leading from reproductive years to permanent infertility that occurs immediately before menopause is termed perimenopause (Dalal and Agarwal, 2015). Perimenopause or “menopause transition” can begin 8–10 years before menopause, when the ovaries gradually produce less estrogen. It usually starts in the fourth decade of woman’s life. In the last 1–2 years of perimenopause, the drop in estrogen accelerates (Figure 1; Dalal and Agarwal, 2015). As a result of a lower level of estrogen, postmenopausal women seem to be at higher risk for a number of diseases, such as osteoporosis, heart disease and dementia (Dalal and Agarwal, 2015).

In males, estradiol is known to be an active metabolic product of testosterone. The serum levels of estradiol in males are about 14–55 pg/ml and are comparable to those of postmenopausal women <35 pg/ml. Testosterone freely enters the brain and might be converted to estradiol by local aromatase enzymes before acting at the cellular level (Gillies and McArthur, 2010). In the central nervous system (CNS) of men and women, specific estrogen receptors (ER) have been shown to localize in mitochondria in the frontal lobe and the hippocampus, confirming an implication of estrogen in controlling memory processes and cognitive functions via energy supply (Genazzani et al., 2007). Further studies have shown that estrogen plays an important neuroprotective role during the aging process, in particular through their beneficial impact upon mitochondrial metabolism (Grimm et al., 2012). Moreover, females live longer than males. This sex difference in life longevity can be attributed in part to the antioxidant effect of estrogen and the up-regulation of life longevity-related genes (Viña et al., 2013).

Normal cellular function can be disrupted by mitochondrial impairment which represents a key feature of the aging process (Balaban et al., 2005; Wanagat et al., 2010). The brain is our most energy-consuming organ and the age-dependent dysregulation in cerebral redox homeostasis and bioenergetics might therefore be able to trigger the development of neurodegenerative disorders.

## Mitochondrial Impairments in Brain Aging: Insight Into the Role of Estrogen

**Figure 2:**
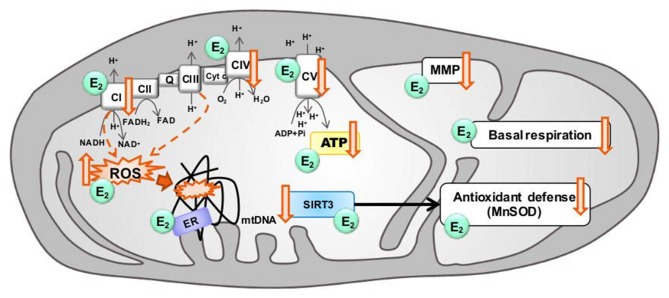
Modulation of mitochondrial function in aging by estrogen. Aging is associated with electron transport chain (ETC) impairments leading to decreased ATP levels, basal respiration and mitochondrial membrane potential (MMP), together with a decrease of antioxidant defenses, and an increase of ROS production by complex I and complex III (dashed arrows) as well as mitochondrial DNA (mtDNA) oxidative damages. Estrogen (E2) has been shown, to increase ETC activity, stabilize the MMP, prevent the ROS production, and ameliorate the basal respiration and the production of ATP levels. Sirtuin 3 (SIRT3) and estrogen may converge on a common pathway to rescue mitochondrial activity in aging by increasing antioxidant defense activity. Estrogen and SIRT3 are suggested to rescue age-related impairments. ↓: age-related decrease, ↑: age-related increase.

Aging is characterized by a progressive decline in physiological functions, often accompanied by neurodegenerative diseases and mitochondrial dysfunction is one of the main factors contributing to the aging process (Figure 2; Cui et al., 2012).

During aging, energy production in the brain is reduced and, in parallel, the redox status is switched from an antioxidant to pro-oxidant state, partly due to the mitochondrial production of O^2−^ and H_2_O_2_ (Yin et al., 2014). Globally, a reduction in mitochondrial functions, including the activity of ETC complexes, the level of the antioxidant glutathione (GSH) as well as the antioxidant defense enzymes such as superoxide dismutase (SOD), was observed with increasing age (Figure 2; Leuner et al., 2012; Grimm et al., 2016a).

We recently reviewed the age-dependent modifications in redox homeostasis and brain bioenergetics, both hallmarks of normal brain aging, with a specific focus on sex differences (Grimm et al., 2016b). We highlighted the tight relationship between the age-related decrease in sex steroid levels and the age-related increase in brain oxidative insults. Especially, women experience a drastic loss of estrogen at the menopause that may be correlated with a decrease of antioxidant defenses (GSH levels) and increased oxidation in the brain (Figure 1; Mandal et al., 2012; Rekkas et al., 2014; Grimm et al., 2016b). Strikingly, women are more armed against oxidative stress before the menopause due to their elevated antioxidant defense mechanisms compared to men (Viña and Borrás, 2010). Healthy young females (mean age: 26 years) showed higher GSH levels in the frontal and parietal cortex compared to young men (Mandal et al., 2012), with a progressive decrease during aging from young to old women (mean age: 56 years). One of the relevant sources of ROS, the monoamine oxidase A (MAO-A) was found to be higher in the brain of perimenopausal women (41–51 years old) compared to young women (21–40 years old; Rekkas et al., 2014). In perimenopausal women who underwent bilateral oophorectomy, the serum estrogen levels were decreased and an increase of oxidative stress was observed. This effect was prevented by a treatment with estrogen (Bellanti et al., 2013).

These human data are supported by numerous animal studies (reviewed in Grimm et al., 2012, 2016b). Namely, Yao et al. (2010) showed that in the brain of perimenopausal mice during reproductive senescence, the drop of estrogen and progesterone (the second female sex hormone) concentrations was accompanied by an increased expression of genes implicated in fatty acid oxidation and the ketogenic pathway, especially at an age between 9 months and 12 months (Figure 1). Further investigations revealed that, in response to a glucose deprivation, the fatty acid metabolism was activated in the perimenopausal mouse brain. This increase was coupled with myelin degeneration as well as a rise of brain ketone bodies that were used as an alternative energy fuel (Figure 1; Yao et al., 2010; Klosinski et al., 2015). Thus, these findings highlighted the role of estrogen on the brain redox balance and energy metabolism at the menopause (Figure 1).

A growing body of evidence supports the mitochondrial theory of aging. The decreased functionality of complex I is often cited as the most likely site of an ETC impairment (Figure 2; Sandhu and Kaur, 2003; Petrosillo et al., 2009; Sun et al., 2016). Indeed, complex I activity is decreased across the brain in aged mice (Pollard et al., 2016), together with a decrease of COX (complex IV) activity and an increase of ROS production (Figure 2; reviewed in Grimm and Eckert, 2017). Finally, modifications in the fatty acid profile of mitochondrial membranes that are more prone to peroxidation are induced by ovariectomy (OVX), and is a feature also reported in aging (Pamplona, 2008).

Mitochondrial DNA (mtDNA) is close to ROS produced by complex I and complex III and seems to be more susceptible to oxidative damage than nuclear DNA (nDNA; Turrens, 2003; Fang et al., 2016). During normal aging, increased levels of oxidative modifications are reported in the brain as well as mutations in mtDNA (Melov, 2004; Vermulst et al., 2007). Of note, no difference in mtDNA oxidative stress markers was detected between 10 months old male and female mice in the liver or the muscle (Sanz et al., 2007). However, since mtDNA is exclusively maternally inherited, female mitochondria appear to be better equipped to prevent mtDNA damage and mutation to reduce the risk of producing inheritable metabolic disorders (reviewed in Demarest and McCarthy, 2015). In line, estrogen supplementation was able to improve the transcription levels of several DNA repair enzymes involved notably in base excision repair pathway in the dorsal raphe of OVX old female macaques (Bethea et al., 2016).

Mitochondrial dynamics play an important role in maintaining a healthy organelle population (Grimm and Eckert, 2017; Schmitt et al., 2018). Impairments in this quality control system lead to the accumulation of defective mitochondria, as well as inefficient mitochondrial transport and distribution. Strikingly, only few studies were focused on mitochondrial fusion/fission activity in the brain during aging (see Grimm and Eckert, 2017). Alterations in the expression of fusion/fission proteins were shown in mice synaptosomal mitochondria, with namely an increase in dynamin-related protein 1 (Drp1) expression, a fission protein, between 5 months and 12 months of age, and a decrease from 12 months to 24 months (Stauch et al., 2014). In parallel, the expression of mitochondrial fusion proteins, including mitofusins 1/2 (Mfn1/2) and optic atrophy 1 (Opa1), decreased from 5 months to 12 months and increased from 12 months to 24 months, suggesting that synaptosomal mitochondria were shifted to a pro-fusion state in aged animals. Additionally, a pro-fusion effect of estrogen (increased mRNA and protein level of Mfn1/2) was shown in MCF-7 breast cancer cell line (Sastre-Serra et al., 2012). However, to our current knowledge, estrogen effects on mitochondrial dynamics in the brain remain still under investigated. An age-related decline in mitophagy (elimination of damaged mitochondria by autophagy) and mitochondrial biogenesis have also been previously reported (reviewed in Grimm and Eckert, 2017), but the precise reason for this decrease remains elusive, especially in the brain. It has been suggested that one of the potential mechanisms regulating mitochondrial biogenesis occurs via hormonal control such as estrogen (reviewed in Scheller and Sekeris, 2003; Chen et al., 2009).

Thus, mitochondrial dysfunction seems to play a central role in brain aging, leading to decreased bioenergetics (glucose metabolism, mitochondrial respiration and ATP synthesis) and increased oxidation (increase of ROS and decrease of antioxidant defenses). High levels of estrogen, especially that of estradiol, before the menopause may be involved in the high energy capacity as well as the control of redox balance in female brain (Figure 1). Then, the drastic drop of estrogen levels may disturb this finely controlled homeostasis, leading to the above-mentioned impairments (see also Grimm et al., 2016b). The Figure 2 summarizes where estrogen can potentially act to prevent or rescue age-related mitochondria impairments (reviewed in Grimm et al., 2012, 2016b see also section: “Estrogen and Bioenergetics in Female Brain: Implications for Estrogen Replacement Therapy”).

## Estrogen in the Central Nervous System

### Mitochondria, Steroid Synthesis and Estrogen

Steroid hormones can be synthesized within the nervous system independently of peripheral steroid glands and are called neurosteroids (Corpéchot et al., 1981). Mitochondria play a crucial role during the first step of the steroid hormone biosynthesis which is the production of pregnenolone, the common precursor of steroids and neurosteroids (Velarde, 2014). The limiting step during steroidogenesis is the transport of cholesterol from the outside to the inside of mitochondria (Papadopoulos and Miller, 2012). This process highlights the paramount role of mitochondria in steroid homeostasis. The interaction between the steroidogenic acute regulatory protein (StAR) and a multi-component molecular complex including an 18 kDa translocator protein (TSPO) regulates the cholesterol transport (Miller, 2013). Once in the mitochondria, cholesterol is converted to pregnenolone by the cytochrome P450 side-chain cleavage enzyme (Miller and Auchus, 2011). Then, pregnenolone can be transferred out of the mitochondria and converted into the different steroids, including estrogen, by specific microsomal P450 enzymes (Miller, 2005).

### Estrogen: From Brain Development to Brain Aging

Today, it is recognized that the action of estrogen is not limited to the regulation of endocrine functions and behavior. Estrogen plays predominant roles as pleiotropic factor in the development and function of the CNS. Numerous evidences highlighted the influence of both male and female sex hormones on neural circuit development, sex-specific behaviors and sexual differentiation of mammalian brain from the fetal-neonatal period to puberty and adult life (reviewed in Hines, 2011). For instance, the transition from childhood to adulthood is marked by morphological changes in the brain that are associated with sex hormone levels (Koolschijn et al., 2014). Interestingly, the structure and function of the brains of female and male animals as well as humans have been demonstrated to be markedly different in the number of cells in specific brain areas (Kruijver et al., 2000). This sexual dimorphism in several areas of the brain appears to be dependent on the action of gonadal hormones as variations in the volume of cortical and sub-cortical regions were correlated to the levels of estrogen and testosterone during puberty (Kruijver et al., 2000; Herting et al., 2014).

Interestingly, the group of Shah showed that the conversion of testosterone to estradiol by the enzyme aromatase is necessary during the perinatal period for the expression of masculine sex behavior in the adult, while the absence of testosterone and estrogen is necessary for normal development of female brains (Wu et al., 2009). This points out the importance of brain estrogen synthesis in aromatase-expressing neurons during fetal male life. Although testosterone is important for male development and behavior throughout life, it is now recognized that estrogen plays also important roles in the male brain (reviewed in Gillies and McArthur, 2010).

Estrogen can induce several effects through different pathways (see also next section). The identification of ER outside their classical CNS regions like the hypothalamus and the pituitary gland justifies their role in controlling different brain functions (Genazzani et al., 2007). This organizational effect is firstly acting during the fetal-neonatal period where estrogen modulates neuronal development and the development of neuronal circuits (Kruijver et al., 2001). Brain plasticity begins with the formation of the nervous system during early development and continues through the puberty, reproduction and adult life (Cooke et al., 1998). Estrogen seems to be also important for the maintenance and regulation of the network integrity of brain areas related to cognition, especially the hippocampus and associated structures (Garcia-Segura et al., 1994). In peri- and postmenopause, neurosteroids including estrogen undergo important changes due to the disturbance of gonadal hormone production and many CNS functions associated with hippocampal activities deteriorate, such as memory, cognition and attention (Genazzani et al., 2005).

### Estrogen Pathways: Estrogen Receptors, BDNF and Sirtuin 3

Different pathways play a role in the pleiotropic effects of estrogen in the brain. In the following, we discuss the emerging evidence about the role of ER and the estrogen—brain-derived neurotrophic factor (BDNF) as well as estrogen—sirtuin 3 (SIRT3) pathways that converge on the mitochondria.

#### Estrogen Receptors

Among the sex steroid hormone receptors, ERα and ERβ have been shown to be localized in mitochondria depending on the cell type and they ensure the mitochondrial function. The co-localization of ERβ and mitochondrial markers have been demonstrated in rat primary neurons and a murine hippocampal cell line suggesting a role of mitochondrial ERβ in mediating estrogen effects (Yang et al., 2004; Figure 2). Indeed, ERβ is described as the type of ER that is most frequently present in mitochondria in most cell types (Vasconsuelo et al., 2013). ERα seems also to play a role, since estrogen exerted protective effects in human cerebral endothelium by increasing mitochondrial cytochrome *c* protein and mRNA, as well as reducing ROS through ERα but not ERβ receptors (Razmara et al., 2008).

In addition, estrogen response elements (ERE) are present in the mtDNA and allow the binding of steroid receptors. In fact, ER can bind to ERE located in the mtDNA (Chen et al., 2004). This association is known to improve the expression of mitochondrial-encoded genes and the ETC activity (Figure 2; Arnold et al., 2012). Moreover, sex steroid hormone receptors have been suggested to bind mitochondrial proteins. Indeed, the association of mitochondrial ERβ with mitochondrial respiratory complex proteins was investigated in a series of co-immunoprecipitation studies showing that ERβ can interact with the respiratory complex V (Alvarez-Delgado et al., 2010; Velarde, 2014). In different brain areas, there is a differential expression of mitochondrial ERα and ERβ variants suggesting that they can participate in several functions in the brain during aging (Alvarez-Delgado et al., 2010). In female rats, mitochondrial ERα and ERβ were detected in the hypothalamus, cortex and hippocampus with no difference between young (3 months old) and aged (18 months old) animals (Alvarez-Delgado et al., 2010).

#### Sirtuin 3 Pathway

The function of the sirtuin family consisting of seven members is important in different aspects of cellular regulation such as lipid homeostasis and metabolism (reviewed Houtkooper et al., 2012), apoptosis resistance (reviewed in Pantazi et al., 2013) and oxidative stress management (reviewed in Rajendran et al., 2011). In addition, sirtuins are believed to contribute to the aging process and may play direct roles in longevity regulation (Guarente, 2013). Among the sirtuin members, SIRT3 has received much attention for its role in aging and neurodegenerative diseases and is reported to affect human lifespan (Reviewed in Ansari et al., 2017). SIRT3 is one of the three genomically expressed sirtuins that localize to mitochondria (Onyango et al., 2002; Schwer et al., 2002) as well as the primary mitochondrial protein deacetylase (Lombard et al., 2007). SIRT3 controls energy demand during stress conditions including oxidative stress conditions related to the aging process (Figure 2; reviewed in Ansari et al., 2017). In addition, its over-expression prevented neuronal derangements in certain *in vitro* and *in vivo* models of aging (Anamika et al., 2017), whereas its knockout in *Sirt3−/−* mouse embryonic fibroblasts induced abnormal mitochondrial physiology as well as increases in stress-induced superoxide levels and increased genomic instability (Kim et al., 2010). At the mRNA level, *Sirt3* is the third most expressed member of the sirtuin family in the whole brain of the adult rat, except in the cerebellum where its expression was modestly lower (Sidorova-Darmos et al., 2014). During the development, mRNA expression pattern of *Sirt3* was similar in cortex, cerebellum and hippocampus, with *Sirt3* mRNA levels being fairly consistent from embryonic day 18 (E18) until 24 months of age (Sidorova-Darmos et al., 2014). Unfortunately, sex differences were not investigated in the study of Sidorova-Darmos et al. (2014), since the sex was not specifically determined for E18, postnatal days (PN) 2, PN7 or PN21 stages, while only female rats were used for the 3 and 24 month tissue samples, but *Sirt3* mRNA levels were similar at least in liver tissue from male and female mice at the age of 11 months (Yu et al., 2015). Braidy et al. (2015) investigated changes in the protein levels of various sirtuins in the aged female rat brain. Twenty-four month old rats showed lower SIRT3 levels in the hippocampus and frontal cortex, but not occipital or temporal lobe, compared to 3 month old rats.

**Figure 3:**
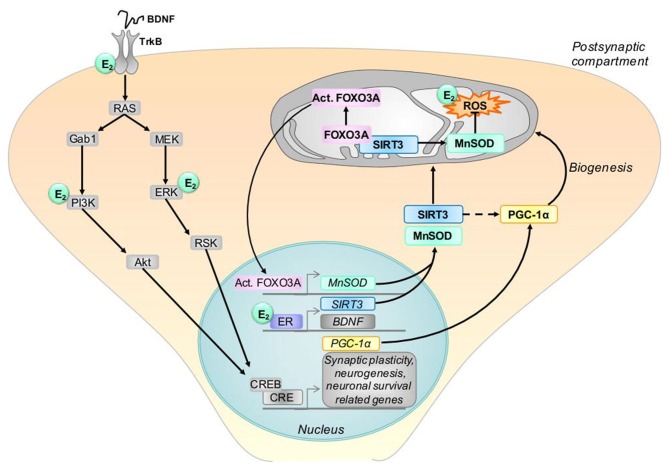
Model of possible interactions between estrogen, BDNF and SIRT3. BDNF protein binds to its tyrosine kinase B receptor (TrkB) which stimulates signaling pathways, including the extracellular signal-regulated kinase (ERK) and the phosphatidylinositol 3-kinase (PI3K) pathways. This leads to the activation of the cAMP response element-binding protein (CREB) and transcription of genes, including peroxisome proliferator-activated receptor gamma coactivator 1-alpha (PGC-1α). Estrogen (E2) activates similar signaling pathways as BDNF, and, by binding its receptor (ER), modulates the gene expression of BDNF, TrkB and SIRT3. SIRT3 protein translocates to mitochondria and interacts with Forkhead box O3 (FOXO3A), thereby activating this protein. In turn, activated FOXO3A (Act. FOXO3A) induces the transcription of the manganese superoxide dismutase (MnSOD) which acts as ROS scavengers in mitochondria. In addition, SIRT3 protects the cell from ROS directly through the deacetylation of MnSOD. SIRT3 regulates also the expression of PGC-1α (indicated by the doted arrow) which is involved in mitochondrial biogenesis. In the graph, E2 designates where estrogen may interact with the BDNF and SIRT3 pathways. BDNF: brain derived neurotrophic factor, CRE, cAMP response elements, PGC-1α, proliferator-activated receptor gamma coactivator 1-alpha, SIRT3, sirtuin 3.

The co-presence of SIRT3 and the peroxisome proliferator-activated receptor-γ coactivator-1α (PGC-1α) implicates its role in mitochondrial biogenesis that provides ATP to support fundamental cellular processes involved in synaptic plasticity such as neurite outgrowth (Kong et al., 2010). Interestingly, the mitochondrial SIRT3 promoter region carries an estrogen-related receptor (ERR)-binding element (ERRE) located 399- to 407-bp downstream (Figure 3; Kong et al., 2010). SIRT3 attenuates ROS and protects the cell from ROS both directly through the deacetylation of manganese superoxide dismutase (MnSOD; Figures 2, 3) and indirectly via interaction with transcription factors such as forkhead box O3 (FOXO3a; Figure 3) as demonstrated in TCam-2 cells (Panza et al., 2017). Sirt3-induced activated FOXO3a translocates to the nucleus and augments FOXO3a-dependent antioxidant defense mechanisms, through upregulation of PGC-1α and MnSOD (Figure 3).

#### BDNF Pathway

Research has implicated one of the neurotrophic factors, BDNF, to be involved in age-related cognitive decline. A hypothesis has emerged that aging is associated with a decreased BDNF signaling capacity in the CNS (Mattson et al., 2004). In humans, BDNF levels have been shown to decrease in brain regions involved in age-related neurodegenerative diseases: e.g., in Alzheimer’s disease (AD) in the frontal cortex (Ferrer et al., 1999), the hippocampus (Phillips et al., 1991), the parietal cortex (Hock et al., 2000) and the entorhinal cortex (Narisawa-Saito et al., 1996). However, age-related changes in brain BDNF levels in elderly humans during normal cognitive aging are under-investigated. In aged monkeys (26, 30 and 32 years), the intensity of BDNF-immunoreactivity has been found to decline in cell bodies and dendrites of the neurons in the hippocampal formation (Hayashi et al., 2001). In humans, BDNF levels in plasma have been found to decrease with increasing age (Lommatzsch et al., 2005; Ziegenhorn et al., 2007; Erickson et al., 2010) and the levels of tropomyosin receptor kinase B (trkB, BNDF receptor) mRNA decreased over the life span (Webster et al., 2006). In accordance with these results, it has been shown that the levels of trkB decrease in the rat hippocampus during aging (Croll et al., 1998; Silhol et al., 2005) and 24-month-old rats exhibited significantly less BDNF protein in three brain regions as compared to 4-month-old rats (Bimonte-Nelson et al., 2008). However, it should be noticed that conflicting data from animal research exist not always confirming whether age-related changes do occur in the brain (Bimonte-Nelson et al., 2008). Compared to females, males had lower BDNF levels in the hippocampus at 20 months emphasizing the important role of sex and sex hormones (Bimonte-Nelson et al., 2008). Therefore, discordant findings between aging studies may also be related to the fact that some studies used males (e.g., Narisawa-Saito and Nawa, 1996; Yurek and Fletcher-Turner, 2001), whereas others used female animals (e.g., Scott et al., 1994). In agreement with these findings, some authors found that elderly females, but not elderly males, with poorer cognitive performance had lower plasma BDNF levels than better performers (e.g., Komulainen et al., 2008) and smaller hippocampal volumes are associated with reduced levels of serum BDNF and poorer memory performance (Erickson et al., 2010). Moreover, genetic studies have identified a single nucleotide polymorphism on the BDNF gene that moderates age-related cognitive decline (Erickson et al., 2008). Of note, a very recent study showed that estrogen tweaks circuit function by interacting with a uniquely human version of the gene that encodes for BDNF, the Val66Met genotype (Wei et al., 2018). The BDNF Val66Met variant and ovarian steroids interactively modulate working memory-related hippocampal function in women suggesting that hormone-gene interaction may underlie sex/individual differences in brain disorders, thus providing a potential explanation for observations of individual differences in the effects of estrogen on hippocampal performance in women (Wei et al., 2018).

In summary, there is evidence suggesting that the BDNF-trkB system, especially in the hippocampus, is sensitive to aging and is modulated by sex and sex hormones respectively. In this context, the drop in estrogen in females during perimenopause might play a role in the cognitive function decline during female aging.

BDNF and estrogen are both important modulators of synaptic plasticity (Figure 3; Zárate et al., 2017). Synaptic plasticity, the dynamic regulation of synaptic mechanisms like long-term potentiation (LTP), spine density and form, number and length of dendrites and axons (neuritogenesis) and the number of neurons (neurogenesis and apoptosis) represent major mechanisms by which our brain can adapt to periods of pathologically enhanced or reduced function or to save information at the synaptic level. Mitochondria play a crucial role as they provide the cellular energy for these adaptive responses or initiate apoptosis in case of neuronal damage beyond the possibility of repair. Changes of synaptic function and plasticity play a major role for cognitive deficits in aging and age-related brain disorders (Barnes, 1990; Rosenzweig and Barnes, 2003).

Activation of the high-affinity BDNF receptor TrkB results in phosphorylation of tyrosine residues in the cytoplasmic domain of the receptor and subsequent recruitment and activation of signaling proteins that engage extracellular signal-regulated kinases (ERKs) and the phosphatidylinositol 3-kinase (PI3K)-Akt kinase pathway (Figure 3; Cheng et al., 2012).

Notably, it has been shown that estrogen and BDNF receptor (trkB) coexpression leads to convergence of their signaling pathways (Figure 3; Scharfman and MacLusky, 2006) and PI3K and MAPK/ERK protein cascades may be activated by estrogen or BDNF-bound membrane receptors (Cover et al., 2014). Both pathways phosphorylate the transcription factor cAMP response element-binding protein (CREB) which, in turn, induces the expression of PGC-1α resulting in protein transcription, neuronal plasticity and memory formation and consolidation (Marosi and Mattson, 2014). Intra-pathway crosstalk is in addition possible. Moreover, estrogen and BDNF can interact directly, because estrogen induces BDNF gene expression, which in turn acts on trkB to exert its effects (Figure 3; Scharfman and MacLusky, 2006). Induction of this kind may occur by an ERE on the BDNF gene or by estrogen-induced increase in neural activity that upregulates BDNF (Figure 3; Scharfman and MacLusky, 2006; Zárate et al., 2017). By enhancing the uptake of energy substrates, mitochondrial biogenesis, and protein synthesis capabilities of neurons, estrogen-BDNF signaling plays pivotal roles in the adaptive changes in synapse formation and modification. Thus, the concerted action between BDNF and estrogen is important for the improvement of synaptic function and plasticity in aging.

## Estrogen and Bioenergetics in Female Brain: Implications for Estrogen Replacement Therapy

Estrogen is an essential regulator of the metabolic system of the female brain and contributes to processes within the entire bioenergetic system including glucose metabolism, glucose transport and ATP production as well as mitochondrial respiration (Figure 2; Rettberg et al., 2014). A decline of 15%–25% in metabolic function in the brain was observed after the loss of estrogen by surgical removal of ovaries or reproductive endocrine aging (Loembe et al., 1988; Slebos et al., 1988). Recent research of our group confirmed *in vitro* the effects of estrogen on mitochondrial function (Figure 2). We showed that estrogen, such as estradiol and estrone, was able to improve bioenergetics and antioxidant defenses in primary neuronal cultures and in human neuroblastoma cells (SH-SY5Y) by increasing mitochondrial membrane potential (MMP), ATP levels, basal respiration and MnSOD activity (Grimm et al., 2014; Figure 2). Besides, a treatment with estrogen was efficient in reducing bioenergetic impairments observed in a cellular model of AD (Grimm et al., 2016a; Lejri et al., 2017). Thus, estrogen represents an attractive therapeutic tool to counteract the mitochondrial impairments in aging and age-related disorders.

Female sex is the major risk factor for AD after advanced age. Preclinical studies demonstrated that the perimenopause to menopause transition is a sex-specific risk factor for AD (Mosconi et al., 2017). In animal studies, estrogenic regulation of cerebral glucose metabolism falters during perimenopause (Mosconi et al., 2017). During aging, a decline of genes required for mitochondrial function and β-amyloid degradation was observed in a rat model recapitulating fundamental characteristics of the human perimenopause (Yin et al., 2015). Impaired synaptic function and emergence of glucose hypometabolism in brain give plausible mechanisms of neurological symptoms of perimenopause and can be predictive of later-life vulnerability to hypometabolic conditions like AD (Yin et al., 2015).

In the metabolic system of the aging female brain, the earliest change is the persistent decline in neuronal glucose transport and metabolism, followed by a drop in mitochondrial function (Ding et al., 2013). In fact, immediately before the transition into reproductive senescence, a hypometabolism in the brain of female mice (at 6–9 months) was demonstrated by a reduction in glucose uptake and hexokinase activity (Ding et al., 2013). These phenomena were also accompanied by inactivation of complex IV activity and pyruvate dehydrogenase leading to an impairment of the mitochondrial energy-conservation system in the brain of female mice (Ding et al., 2013). In parallel to the decline in glucose transport, lactate transport and utilization were also reduced, suggesting that the lactate is not used as an alternative fuel source during the transition to reproductive senescence.

Earlier preclinical findings indicating emergence of bioenergetic deficits in perimenopausal and postmenopausal women were recently validated and suggested that the optimal window of opportunity for therapeutic intervention in women is early in the endocrine aging process (Mosconi et al., 2017). A study bridged basic to clinical science to characterize brain bioenergetics in a cohort of 43, 40–60 year-old clinically and cognitively normal women at different endocrine transition stages including premenopause, perimenopause and postmenopause. Compared to premenopause, both perimenopause and postmenopause groups exhibited a reduction in mitochondrial COX activity which was correlated with a decline of cerebral glucose metabolism in AD-vulnerable regions. A gradient in biomarker abnormalities correlated with immediate and delayed memory scores and was most pronounced in post-menopause, intermediate in perimenopause, and lowest in premenopause (Mosconi et al., 2017). Therefore, estrogen replacement therapies (ERT) may only be beneficial before women accumulate a certain threshold of cellular damage, at the appropriate time of a woman’s life, also known as the “critical period” that starts during perimenopause and ends early after menopause (Figure 1; Velarde, 2013; Viña et al., 2013; Grimm et al., 2016b). This “critical period” might also explain the failure of the Women’s Health Initiative (WHI) study including only postmenopausal aged over 68 years thereby missing the vulnerable time frame (Rossouw et al., 2002; Anderson et al., 2004). The window of opportunity concept supports that estrogen can have beneficial effects only on healthy brain during the years before the menopause compared to some years after the menopause onset where ERT could even present detrimental effect on brain activity (Morrison et al., 2006; Brinton, 2008, 2009; Scott et al., 2012; Rettberg et al., 2014; Miller and Harman, 2017). The effectiveness of ERT is also dependent on the formulation of treatments (e.g., use of synthetic conjugated equine estrogen vs. natural estrogen, or co-treatment with progesterone), the mode of delivery (transdermal or oral) and the regimen (cyclic or continuous; Miller and Harman, 2017).

**Figure 4:**
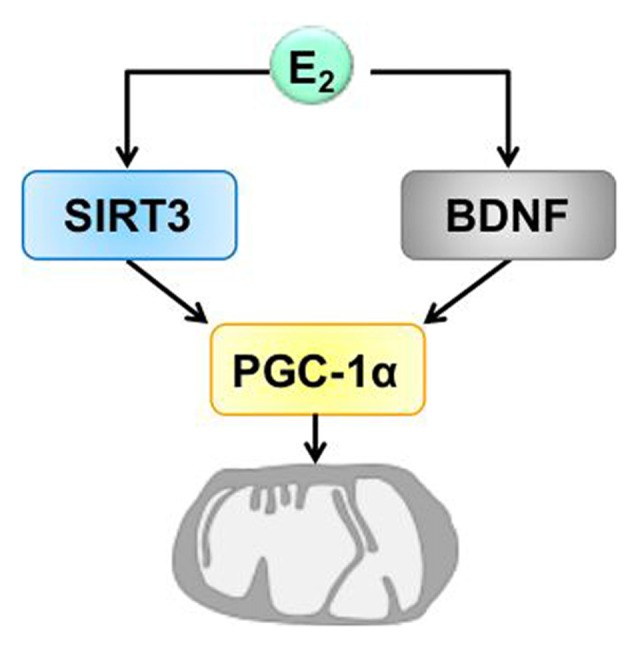
Hypothetic model of the convergence of BDNF and SIRT3 on mitochondria induced by estrogen. Estrogen (E2) induces the expression of BDNF and SIRT3. The action of BDNF and SIRT3 converges on mitochondria by regulating the expression of PGC-1α which is involved in mitochondrial biogenesis that is necessary for synaptic plasticity. BDNF, brain derived neurotrophic factor, PGC-1α, proliferator-activated receptor gamma coactivator 1-alpha, SIRT3, sirtuin 3.

Importantly, recent evaluation of the role of estrogen showed that hormonal therapy can prevent the deleterious effects of aging in cognition, and decreases the risks of dementia, if initiated early (Girard et al., 2017). In this context, beneficial treatment effects of estrogen might be mediated by the BDNF and SIRT3 pathways and their convergence via PGC-1α on mitochondria (Figure 4).

## Conclusion

Over the last decade, accumulating evidence has suggested a causative link between mitochondrial dysfunction and major phenotypes associated with aging. A number of recent studies link mitochondrial function to signaling pathways that regulate brain plasticity, life span and to the aging process. It is clear that estrogen exerts actions on the mitochondria and that these organelles play an important role in age-related processes. In this context, estrogen/BDNF or estrogen/SIRT3 actions and interactions represent complex and fundamental mechanisms of neuronal plasticity, a process highly depending on energy supply via mitochondrial activity (Figure 4). Notably, a growing body of evidence indicates emergence of bioenergetic deficits already in perimenopausal women, suggesting that the optimal window of opportunity should be used for the therapeutic intervention in women during the endocrine aging process.

A full understanding of the molecular mechanisms triggered by estrogen at the mitochondrial level and its effects in aging populations may provide profound and exciting possibilities for the future treatment of age-dependent diseases associated with the deregulation of sexual hormone levels.

## Author Contributions

The authors had an equal contribution in the writing of the manuscript (IL, AG and AE).

## Conflict of Interest Statement

The authors declare that the research was conducted in the absence of any commercial or financial relationships that could be construed as a potential conflict of interest.

## References

[B1] Alvarez-Delgado C. Mendoza-Rodríguez C. A. Picazo O. Cerbón M. (2010). Different expression of α and β mitochondrial estrogen receptors in the aging rat brain: interaction with respiratory complex V. Exp. Gerontol. 45, 580–585. 10.1016/j.exger.2010.01.015 20096765

[B2] Anamika Khanna A. Acharjee P. Acharjee A. Trigun S. K. (2017). Mitochondrial SIRT3 and neurodegenerative brain disorders. J. Chem. Neuroanat. [Epub ahead of print]. 10.1016/j.jchemneu.2017.11.009 29129747

[B3] Anderson G. L. Limacher M. Assaf A. R. Bassford T. Beresford S. A. Black H.. (2004). Effects of conjugated equine estrogen in postmenopausal women with hysterectomy: the Women’s health initiative randomized controlled trial. JAMA 291, 1701–1712. 10.1001/jama.291.14.1701 15082697

[B4] Ansari A. Rahman M. S. Saha S. K. Saikot F. K. Deep A. Kim K. H. (2017). Function of the SIRT3 mitochondrial deacetylase in cellular physiology, cancer, and neurodegenerative disease. Aging Cell 16, 4–16. 10.1111/acel.12538 27686535 PMC5242307

[B5] Arnold S. Victor M. B. Beyer C. (2012). Estrogen and the regulation of mitochondrial structure and function in the brain. J. Steroid Biochem. Mol. Biol. 131, 2–9. 10.1016/j.jsbmb.2012.01.012 22326731

[B6] Balaban R. S. Nemoto S. Finkel T. (2005). Mitochondria, oxidants, and aging. Cell 120, 483–495. 10.1016/j.cell.2005.02.001 15734681

[B7] Barnes C. A. (1990). Effects of aging on the dynamics of information processing and synaptic weight changes in the mammalian hippocampus. Prog. Brain Res. 86, 89–104. 10.1016/s0079-6123(08)63169-6 1965057

[B8] Bellanti F. Matteo M. Rollo T. De Rosario F. Greco P. Vendemiale G.. (2013). Sex hormones modulate circulating antioxidant enzymes: impact of estrogen therapy. Redox Biol. 1, 340–346. 10.1016/j.redox.2013.05.003 24024169 PMC3757703

[B9] Bethea C. L. Kohama S. G. Reddy A. P. Urbanski H. F. (2016). Ovarian steroids regulate gene expression in the dorsal raphe of old female macaques. Neurobiol. Aging 37, 179–191. 10.1016/j.neurobiolaging.2015.10.004 26686671 PMC4699313

[B10] Bimonte-Nelson H. A. Granholm A. C. Nelson M. E. Moore A. B. (2008). Patterns of neurotrophin protein levels in male and female Fischer 344 rats from adulthood to senescence: how young is “young” and how old is “old”? Exp. Aging Res. 34, 13–26. 10.1080/03610730701761908 18189165 PMC2692474

[B11] Braidy N. Poljak A. Grant R. Jayasena T. Mansour H. Chan-Ling T.. (2015). Differential expression of sirtuins in the aging rat brain. Front. Cell. Neurosci. 9:167. 10.3389/fncel.2015.00167 26005404 PMC4424846

[B12] Brinton R. D. (2008). The healthy cell bias of estrogen action: mitochondrial bioenergetics and neurological implications. Trends Neurosci. 31, 529–537. 10.1016/j.tins.2008.07.003 18774188 PMC10124615

[B13] Brinton R. D. (2009). Estrogen-induced plasticity from cells to circuits: predictions for cognitive function. Trends Pharmacol. Sci. 30, 212–222. 10.1016/j.tips.2008.12.006 19299024 PMC3167490

[B14] Castellani R. J. Rolston R. K. Smith M. A. (2010). Alzheimer disease. Dis. Mon. 56, 484–546. 10.1016/j.disamonth.2010.06.001 20831921 PMC2941917

[B15] Chen J. Q. Cammarata P. R. Baines C. P. Yager J. D. (2009). Regulation of mitochondrial respiratory chain biogenesis by estrogens/estrogen receptors and physiological, pathological and pharmacological implications. Biochim. Biophys. Acta 1793, 1540–1570. 10.1016/j.bbamcr.2009.06.001 19559056 PMC2744640

[B16] Chen J. Q. Eshete M. Alworth W. L. Yager J. D. (2004). Binding of MCF-7 cell mitochondrial proteins and recombinant human estrogen receptors α and β to human mitochondrial DNA estrogen response elements. J. Cell. Biochem. 93, 358–373. 10.1002/jcb.20178 15368362

[B17] Cheng A. Wan R. Yang J. L. Kamimura N. Son T. G. Ouyang X.. (2012). Involvement of PGC-1α in the formation and maintenance of neuronal dendritic spines. Nat. Commun. 3:1250. 10.1038/ncomms2238 23212379 PMC4091730

[B18] Cooke B. Hegstrom C. D. Villeneuve L. S. Breedlove S. M. (1998). Sexual differentiation of the vertebrate brain: principles and mechanisms. Front. Neuroendocrinol. 19, 323–362. 10.1006/frne.1998.0171 9799588

[B19] Corpéchot C. Robel P. Axelson M. Sjövall J. Baulieu E. E. (1981). Characterization and measurement of dehydroepiandrosterone sulfate in rat brain. Proc. Natl. Acad. Sci. U S A 78, 4704–4707. 10.1073/pnas.78.8.4704 6458035 PMC320231

[B20] Cover K. K. Maeng L. Y. Lebrón-Milad K. Milad M. R. (2014). Mechanisms of estradiol in fear circuitry: implications for sex differences in psychopathology. Transl. Psychiatry 4:e422. 10.1038/tp.2014.67 25093600 PMC4150242

[B21] Croll S. D. Ip N. Y. Lindsay R. M. Wiegand S. J. (1998). Expression of BDNF and trkB as a function of age and cognitive performance. Brain Res. 812, 200–208. 10.1016/s0006-8993(98)00993-7 9813325

[B22] Cui H. Kong Y. Zhang H. (2012). Oxidative stress, mitochondrial dysfunction, and aging. J. Signal Transduct. 2012:646354. 10.1016/b978-0-12-405933-7.00004-4 21977319 PMC3184498

[B23] Dalal P. K. Agarwal M. (2015). Postmenopausal syndrome. Indian J. Psychiatry 57, S222–S232. 10.4103/0019-5545.161483 26330639 PMC4539866

[B24] Demarest T. G. McCarthy M. M. (2015). Sex differences in mitochondrial (dys)function: implications for neuroprotection. J. Bioenerg. Biomembr. 47, 173–188. 10.1007/s10863-014-9583-7 25293493 PMC4988325

[B25] Ding F. Yao J. Rettberg J. R. Chen S. Brinton R. D. (2013). Early decline in glucose transport and metabolism precedes shift to ketogenic system in female aging and Alzheimer’s mouse brain: implication for bioenergetic intervention. PLoS One 8:e79977. 10.1371/journal.pone.0079977 24244584 PMC3823655

[B26] Erickson K. I. Kim J. S. Suever B. L. Voss M. W. Francis B. M. Kramer A. F. (2008). Genetic contributions to age-related decline in executive function: a 10-year longitudinal study of COMT and BDNF polymorphisms. Front. Hum. Neurosci. 2:11. 10.3389/neuro.09.009.2008 18958211 PMC2572207

[B27] Erickson K. I. Prakash R. S. Voss M. W. Chaddock L. Heo S. Mclaren M.. (2010). Brain-derived neurotrophic factor is associated with age-related decline in hippocampal volume. J. Neurosci. 30, 5368–5375. 10.1523/jneurosci.6251-09.2010 20392958 PMC3069644

[B28] Fang E. F. Scheibye-Knudsen M. Chua K. F. Mattson M. P. Croteau D. L. Bohr V. A. (2016). Nuclear DNA damage signalling to mitochondria in ageing. Nat. Rev. Mol. Cell Biol. 17, 308–321. 10.1038/nrm.2016.14 26956196 PMC5161407

[B29] Ferrer I. Marín C. Rey M. J. Ribalta T. Goutan E. Blanco R.. (1999). BDNF and full-length and truncated TrkB expression in Alzheimer disease. Implications in therapeutic strategies. J. Neuropathol. Exp. Neurol. 58, 729–739. 10.1097/00005072-199907000-00007 10411343

[B30] Garcia-Segura L. M. Chowen J. A. Duenas M. Torres-Aleman I. Naftolin F. (1994). Gonadal steroids as promoters of neuro-glial plasticity. Psychoneuroendocrinology 19, 445–453. 10.1016/0306-4530(94)90031-0 7938345

[B31] Genazzani A. R. Bernardi F. Pluchino N. Begliuomini S. Lenzi E. Casarosa E.. (2005). Endocrinology of menopausal transition and its brain implications. CNS Spectr. 10, 449–457. 10.1017/s1092852900023142 15908899

[B32] Genazzani A. R. Pluchino N. Luisi S. Luisi M. (2007). Estrogen, cognition and female ageing. Hum. Reprod. Update 13, 175–187. 10.1093/humupd/dml042 17135285

[B33] Gillies G. E. McArthur S. (2010). Estrogen actions in the brain and the basis for differential action in men and women: a case for sex-specific medicines. Pharmacol. Rev. 62, 155–198. 10.1124/pr.109.002071 20392807 PMC2879914

[B34] Girard R. Météreau E. Thomas J. Pugeat M. Qu C. Dreher J. C. (2017). Hormone therapy at early post-menopause increases cognitive control-related prefrontal activity. Sci. Rep. 7:44917. 10.1038/srep44917 28322310 PMC5359606

[B35] Grimm A. Eckert A. (2017). Brain aging and neurodegeneration: from a mitochondrial point of view. J. Neurochem. 143, 418–431. 10.1111/jnc.14037 28397282 PMC5724505

[B36] Grimm A. Friedland K. Eckert A. (2016a). Mitochondrial dysfunction: the missing link between aging and sporadic Alzheimer’s disease. Biogerontology 17, 281–296. 10.1007/s10522-015-9618-4 26468143

[B38] Grimm A. Mensah-Nyagan A. G. Eckert A. (2016b). Alzheimer, mitochondria and gender. Neurosci. Biobehav. Rev. 67, 89–101. 10.1016/j.neubiorev.2016.04.012 27139022

[B37] Grimm A. Lim Y. A. Mensah-Nyagan A. G. Götz J. Eckert A. (2012). Alzheimer’s disease, oestrogen and mitochondria: an ambiguous relationship. Mol. Neurobiol. 46, 151–160. 10.1007/s12035-012-8281-x 22678467 PMC3443477

[B39] Grimm A. Schmitt K. Lang U. E. Mensah-Nyagan A. G. Eckert A. (2014). Improvement of neuronal bioenergetics by neurosteroids: implications for age-related neurodegenerative disorders. Biochim. Biophys. Acta 1842, 2427–2438. 10.1016/j.bbadis.2014.09.013 25281013

[B40] Guarente L. (2013). Calorie restriction and sirtuins revisited. Genes Dev. 27, 2072–2085. 10.1101/gad.227439.113 24115767 PMC3850092

[B41] Hayashi M. Mistunaga F. Ohira K. Shimizu K. (2001). Changes in BDNF-immunoreactive structures in the hippocampal formation of the aged macaque monkey. Brain Res. 918, 191–196. 10.1016/s0006-8993(01)03002-5 11684059

[B42] Herting M. M. Gautam P. Spielberg J. M. Kan E. Dahl R. E. Sowell E. R. (2014). The role of testosterone and estradiol in brain volume changes across adolescence: a longitudinal structural MRI study. Hum. Brain Mapp. 35, 5633–5645. 10.1002/hbm.22575 24977395 PMC4452029

[B43] Hines M. (2011). Prenatal endocrine influences on sexual orientation and on sexually differentiated childhood behavior. Front. Neuroendocrinol. 32, 170–182. 10.1016/j.yfrne.2011.02.006 21333673 PMC3296090

[B44] Hock C. Heese K. Hulette C. Rosenberg C. Otten U. (2000). Region-specific neurotrophin imbalances in Alzheimer disease: decreased levels of brain-derived neurotrophic factor and increased levels of nerve growth factor in hippocampus and cortical areas. Arch. Neurol. 57, 846–851. 10.1001/archneur.57.6.846 10867782

[B45] Houtkooper R. H. Pirinen E. Auwerx J. (2012). Sirtuins as regulators of metabolism and healthspan. Nat. Rev. Mol. Cell Biol. 13, 225–238. 10.1038/nrm3293 22395773 PMC4872805

[B46] Kim H. S. Patel K. Muldoon-Jacobs K. Bisht K. S. Aykin-Burns N. Pennington J. D.. (2010). SIRT3 is a mitochondria-localized tumor suppressor required for maintenance of mitochondrial integrity and metabolism during stress. Cancer Cell 17, 41–52. 10.1016/j.ccr.2009.11.023 20129246 PMC3711519

[B47] Klosinski L. P. Yao J. Yin F. Fonteh A. N. Harrington M. G. Christensen T. A.. (2015). White matter lipids as a ketogenic fuel supply in aging female brain: implications for Alzheimer’s disease. EBioMedicine 2, 1888–1904. 10.1016/j.ebiom.2015.11.002 26844268 PMC4703712

[B48] Komulainen P. Pedersen M. Hänninen T. Bruunsgaard H. Lakka T. A. Kivipelto M.. (2008). BDNF is a novel marker of cognitive function in ageing women: the DR’s EXTRA study. Neurobiol. Learn. Mem. 90, 596–603. 10.1016/j.nlm.2008.07.014 18707012

[B49] Kong X. Wang R. Xue Y. Liu X. Zhang H. Chen Y.. (2010). Sirtuin 3, a new target of PGC-1α, plays an important role in the suppression of ROS and mitochondrial biogenesis. PLoS One 5:e11707. 10.1371/journal.pone.0011707 20661474 PMC2908542

[B50] Koolschijn P. C. Peper J. S. Crone E. A. (2014). The influence of sex steroids on structural brain maturation in adolescence. PLoS One 9:e83929. 10.1371/journal.pone.0083929 24416184 PMC3885531

[B51] Kruijver F. P. Fernández-Guasti A. Fodor M. Kraan E. M. Swaab D. F. (2001). Sex differences in androgen receptors of the human mamillary bodies are related to endocrine status rather than to sexual orientation or transsexuality. J. Clin. Endocrinol. Metab. 86, 818–827. 10.1210/jcem.86.2.7258 11158052

[B52] Kruijver F. P. Zhou J. N. Pool C. W. Hofman M. A. Gooren L. J. Swaab D. F. (2000). Male-to-female transsexuals have female neuron numbers in a limbic nucleus. J. Clin. Endocrinol. Metab. 85, 2034–2041. 10.1210/jc.85.5.2034 10843193

[B53] Lejri I. Grimm A. Miesch M. Geoffroy P. Eckert A. Mensah-Nyagan A. G. (2017). Allopregnanolone and its analog BR 297 rescue neuronal cells from oxidative stress-induced death through bioenergetic improvement. Biochim. Biophys. Acta 1863, 631–642. 10.1016/j.bbadis.2016.12.007 27979708

[B54] Leuner K. Müller W. E. Reichert A. S. (2012). From mitochondrial dysfunction to amyloid β formation: novel insights into the pathogenesis of Alzheimer’s disease. Mol. Neurobiol. 46, 186–193. 10.1007/s12035-012-8307-4 22833458

[B55] Loembe P. M. Bouger D. Dukuly L. (1988). Injuries of the cervical spine. Review of 70 cases treated over a 5-year period at the “Fondation Jeanne Ebori” of Libreville, Gabon (Central Africa). Neurochirurgie 34, 258–261. 3200365

[B56] Lombard D. B. Alt F. W. Cheng H. L. Bunkenborg J. Streeper R. S. Mostoslavsky R.. (2007). Mammalian Sir2 homolog SIRT3 regulates global mitochondrial lysine acetylation. Mol. Cell. Biol. 27, 8807–8814. 10.1128/mcb.01636-07 17923681 PMC2169418

[B57] Lommatzsch M. Zingler D. Schuhbaeck K. Schloetcke K. Zingler C. Schuff-Werner P.. (2005). The impact of age, weight and gender on BDNF levels in human platelets and plasma. Neurobiol. Aging 26, 115–123. 10.1016/j.neurobiolaging.2004.03.002 15585351

[B58] Mandal P. K. Tripathi M. Sugunan S. (2012). Brain oxidative stress: detection and mapping of anti-oxidant marker ‘Glutathione’ in different brain regions of healthy male/female, MCI and Alzheimer patients using non-invasive magnetic resonance spectroscopy. Biochem. Biophys. Res. Commun. 417, 43–48. 10.1016/j.bbrc.2011.11.047 22120629

[B59] Marosi K. Mattson M. P. (2014). BDNF mediates adaptive brain and body responses to energetic challenges. Trends Endocrinol. Metab. 25, 89–98. 10.1016/j.tem.2013.10.006 24361004 PMC3915771

[B60] Mattson M. P. Maudsley S. Martin B. (2004). BDNF and 5-HT: a dynamic duo in age-related neuronal plasticity and neurodegenerative disorders. Trends Neurosci. 27, 589–594. 10.1016/j.tins.2004.08.001 15374669

[B61] Melov S. (2004). Modeling mitochondrial function in aging neurons. Trends Neurosci. 27, 601–606. 10.1016/j.tins.2004.08.004 15374671

[B64] Miller W. L. (2005). Minireview: regulation of steroidogenesis by electron transfer. Endocrinology 146, 2544–2550. 10.1210/en.2005-0096 15774560

[B65] Miller W. L. (2013). Steroid hormone synthesis in mitochondria. Mol. Cell. Endocrinol. 379, 62–73. 10.1016/j.mce.2013.04.014 23628605

[B63] Miller W. L. Auchus R. J. (2011). The molecular biology, biochemistry, and physiology of human steroidogenesis and its disorders. Endocr Rev 32, 81–151. 10.1210/er.2010-0013 21051590 PMC3365799

[B62] Miller V. M. Harman S. M. (2017). An update on hormone therapy in postmenopausal women: mini-review for the basic scientist. Am. J. Physiol. Heart Circ. Physiol. 313, H1013–H1021. 10.1152/ajpheart.00383.2017 28801526 PMC5792205

[B66] Morrison J. H. Brinton R. D. Schmidt P. J. Gore A. C. (2006). Estrogen, menopause, and the aging brain: how basic neuroscience can inform hormone therapy in women. J. Neurosci. 26, 10332–10348. 10.1523/JNEUROSCI.3369-06.2006 17035515 PMC6674699

[B67] Mosconi L. Berti V. Guyara-Quinn C. McHugh P. Petrongolo G. Osorio R. S.. (2017). Perimenopause and emergence of an Alzheimer’s bioenergetic phenotype in brain and periphery. PLoS One 12:e0185926. 10.1371/journal.pone.0185926 29016679 PMC5634623

[B68] Narisawa-Saito M. Nawa H. (1996). Differential regulation of hippocampal neurotrophins during aging in rats. J. Neurochem. 67, 1124–1131. 10.1046/j.1471-4159.1996.67031124.x 8752119

[B69] Narisawa-Saito M. Wakabayashi K. Tsuji S. Takahashi H. Nawa H. (1996). Regional specificity of alterations in NGF, BDNF and NT-3 levels in Alzheimer’s disease. Neuroreport 7, 2925–2928. 10.1097/00001756-199611250-00024 9116211

[B70] Onyango P. Celic I. Mccaffery J. M. Boeke J. D. Feinberg A. P. (2002). SIRT3, a human SIR2 homologue, is an NAD-dependent deacetylase localized to mitochondria. Proc. Natl. Acad. Sci. U S A 99, 13653–13658. 10.1073/pnas.222538099 12374852 PMC129731

[B71] Pamplona R. (2008). Membrane phospholipids, lipoxidative damage and molecular integrity: a causal role in aging and longevity. Biochim. Biophys. Acta 1777, 1249–1262. 10.1016/j.bbabio.2008.07.003 18721793

[B72] Pantazi E. Zaouali M. A. Bejaoui M. Folch-Puy E. Ben Abdennebi H. Rosello-Catafau J. (2013). Role of sirtuins in ischemia-reperfusion injury. World J. Gastroenterol. 19, 7594–7602. 10.3748/wjg.v19.i43.7594 24616566 PMC3837258

[B73] Panza S. Santoro M. De Amicis F. Morelli C. Passarelli V. D’Aquila P.. (2017). Estradiol via estrogen receptor β influences ROS levels through the transcriptional regulation of SIRT3 in human seminoma TCam-2 cells. Tumour Biol. 39:1010428317701642. 10.1177/1010428317701642 28459202

[B74] Papadopoulos V. Miller W. L. (2012). Role of mitochondria in steroidogenesis. Best Pract. Res. Clin. Endocrinol. Metab. 26, 771–790. 10.1016/j.beem.2012.05.002 23168279

[B75] Petrosillo G. Matera M. Moro N. Ruggiero F. M. Paradies G. (2009). Mitochondrial complex I dysfunction in rat heart with aging: critical role of reactive oxygen species and cardiolipin. Free Radic. Biol. Med. 46, 88–94. 10.1016/j.freeradbiomed.2008.09.031 18973802

[B76] Phillips H. S. Hains J. M. Armanini M. Laramee G. R. Johnson S. A. Winslow J. W. (1991). BDNF mRNA is decreased in the hippocampus of individuals with Alzheimer’s disease. Neuron 7, 695–702. 10.1016/0896-6273(91)90273-3 1742020

[B77] Pollard A. K. Craig E. L. Chakrabarti L. (2016). Mitochondrial complex 1 activity measured by spectrophotometry is reduced across all brain regions in ageing and more specifically in neurodegeneration. PLoS One 11:e0157405. 10.1371/journal.pone.0157405 27333203 PMC4917223

[B78] Quinlan C. L. Perevoshchikova I. V. Hey-Mogensen M. Orr A. L. Brand M. D. (2013). Sites of reactive oxygen species generation by mitochondria oxidizing different substrates. Redox Biol. 1, 304–312. 10.1016/j.redox.2013.04.005 24024165 PMC3757699

[B79] Rajendran R. Garva R. Krstic-Demonacos M. Demonacos C. (2011). Sirtuins: molecular traffic lights in the crossroad of oxidative stress, chromatin remodeling, and transcription. J. Biomed. Biotechnol. 2011:368276. 10.1155/2011/368276 21912480 PMC3168296

[B80] Razmara A. Sunday L. Stirone C. Wang X. B. Krause D. N. Duckles S. P.. (2008). Mitochondrial effects of estrogen are mediated by estrogen receptor α in brain endothelial cells. J. Pharmacol. Exp. Ther. 325, 782–790. 10.1124/jpet.107.134072 18354059 PMC2650426

[B81] Rekkas P. V. Wilson A. A. Lee V. W. Yogalingam P. Sacher J. Rusjan P.. (2014). Greater monoamine oxidase a binding in perimenopausal age as measured with carbon 11-labeled harmine positron emission tomography. JAMA Psychiatry 71, 873–879. 10.1001/jamapsychiatry.2014.250 24898155 PMC4942269

[B82] Rettberg J. R. Yao J. Brinton R. D. (2014). Estrogen: a master regulator of bioenergetic systems in the brain and body. Front. Neuroendocrinol. 35, 8–30. 10.1016/j.yfrne.2013.08.001 23994581 PMC4024050

[B83] Rizzuto R. De Stefani D. Raffaello A. Mammucari C. (2012). Mitochondria as sensors and regulators of calcium signalling. Nat. Rev. Mol. Cell Biol. 13, 566–578. 10.1038/nrm3412 22850819

[B84] Rosenzweig E. S. Barnes C. A. (2003). Impact of aging on hippocampal function: plasticity, network dynamics, and cognition. Prog. Neurobiol. 69, 143–179. 10.1016/s0301-0082(02)00126-0 12758108

[B85] Rossouw J. E. Anderson G. L. Prentice R. L. LaCroix A. Z. Kooperberg C. Stefanick M. L.. (2002). Risks and benefits of estrogen plus progestin in healthy postmenopausal women: principal results From the Women’s Health Initiative randomized controlled trial. JAMA 288, 321–333. 10.1016/s1062-1458(02)00919-4 12117397

[B86] Sandhu S. K. Kaur G. (2003). Mitochondrial electron transport chain complexes in aging rat brain and lymphocytes. Biogerontology 4, 19–29. 10.1023/A:1022473219044 12652186

[B87] Sanz A. Hiona A. Kujoth G. C. Seo A. Y. Hofer T. Kouwenhoven E.. (2007). Evaluation of sex differences on mitochondrial bioenergetics and apoptosis in mice. Exp. Gerontol. 42, 173–182. 10.1016/j.exger.2006.10.003 17118599 PMC1817668

[B88] Sastre-Serra J. Nadal-Serrano M. Pons D. G. Roca P. Oliver J. (2012). Mitochondrial dynamics is affected by 17β-estradiol in the MCF-7 breast cancer cell line. Effects on fusion and fission related genes. Int. J. Biochem. Cell Biol. 44, 1901–1905. 10.1016/j.biocel.2012.07.012 22824300

[B89] Scharfman H. E. MacLusky N. J. (2006). Estrogen and brain-derived neurotrophic factor (BDNF) in hippocampus: complexity of steroid hormone-growth factor interactions in the adult CNS. Front. Neuroendocrinol. 27, 415–435. 10.1016/j.yfrne.2006.09.004 17055560 PMC1778460

[B90] Scheller K. Sekeris C. E. (2003). The effects of steroid hormones on the transcription of genes encoding enzymes of oxidative phosphorylation. Exp. Physiol. 88, 129–140. 10.1113/eph8802507 12525861

[B91] Schmitt K. Grimm A. Dallmann R. Oettinghaus B. Restelli L. M. Witzig M.. (2018). Circadian control of DRP1 activity regulates mitochondrial dynamics and bioenergetics. Cell Metab. 27, 657.e5–666.e5. 10.1016/j.cmet.2018.01.011 29478834

[B92] Schwer B. North B. J. Frye R. A. Ott M. Verdin E. (2002). The human silent information regulator (Sir)2 homologue hSIRT3 is a mitochondrial nicotinamide adenine dinucleotide-dependent deacetylase. J. Cell Biol. 158, 647–657. 10.1083/jcb.200205057 12186850 PMC2174009

[B94] Scott S. A. Liang S. Weingartner J. A. Crutcher K. A. (1994). Increased NGF-like activity in young but not aged rat hippocampus after septal lesions. Neurobiol. Aging 15, 337–346. 10.1016/0197-4580(94)90029-9 7936058

[B93] Scott E. Zhang Q. G. Wang R. Vadlamudi R. Brann D. (2012). Estrogen neuroprotection and the critical period hypothesis. Front. Neuroendocrinol. 33, 85–104. 10.1016/j.yfrne.2011.10.001 22079780 PMC3288697

[B95] Sidorova-Darmos E. Wither R. G. Shulyakova N. Fisher C. Ratnam M. Aarts M.. (2014). Differential expression of sirtuin family members in the developing, adult, and aged rat brain. Front. Aging Neurosci. 6:333. 10.3389/fnagi.2014.00333 25566066 PMC4270178

[B96] Silhol M. Bonnichon V. Rage F. Tapia-Arancibia L. (2005). Age-related changes in brain-derived neurotrophic factor and tyrosine kinase receptor isoforms in the hippocampus and hypothalamus in male rats. Neuroscience 132, 613–624. 10.1016/j.neuroscience.2005.01.008 15837123

[B97] Slebos R. J. de Graeff A. Van Zandwijk N. Mooi W. J. Bos J. L. Rodenhuis S. (1988). Recurrent breast cancer and an adenocarcinoma of the lung occurring in one patient: c-myc oncogene amplification and K-ras codon 12 point mutation as tumour markers. Eur. J. Cancer Clin. Oncol. 24, 1529–1530. 10.1016/0277-5379(88)90347-1 3181273

[B98] Stauch K. L. Purnell P. R. Fox H. S. (2014). Aging synaptic mitochondria exhibit dynamic proteomic changes while maintaining bioenergetic function. Aging 6, 320–334. 10.18632/aging.100657 24827396 PMC4032798

[B99] Sun N. Youle R. J. Finkel T. (2016). The mitochondrial basis of aging. Mol. Cell 61, 654–666. 10.1016/j.molcel.2016.01.028 26942670 PMC4779179

[B100] Turrens J. F. (2003). Mitochondrial formation of reactive oxygen species. J. Physiol. 552, 335–344. 10.1113/jphysiol.2003.049478 14561818 PMC2343396

[B101] Vasconsuelo A. Milanesi L. Boland R. (2013). Actions of 17β-estradiol and testosterone in the mitochondria and their implications in aging. Ageing Res. Rev. 12, 907–917. 10.1016/j.arr.2013.09.001 24041489

[B102] Velarde M. C. (2013). Reply to turner and kerber. Physiol. Genomics 45:448. 10.1152/physiolgenomics.00053.2013 23733806

[B103] Velarde M. C. (2014). Mitochondrial and sex steroid hormone crosstalk during aging. Longev. Healthspan 3:2. 10.1186/2046-2395-3-2 24495597 PMC3922316

[B104] Vermulst M. Bielas J. H. Kujoth G. C. Ladiges W. C. Rabinovitch P. S. Prolla T. A.. (2007). Mitochondrial point mutations do not limit the natural lifespan of mice. Nat. Genet. 39, 540–543. 10.1038/ng1988 17334366

[B105] Vest R. S. Pike C. J. (2013). Gender, sex steroid hormones, and Alzheimer’s disease. Horm. Behav. 63, 301–307. 10.1016/j.yhbeh.2012.04.006 22554955 PMC3413783

[B106] Viña J. Borrás C. (2010). Women live longer than men: understanding molecular mechanisms offers opportunities to intervene by using estrogenic compounds. Antioxid. Redox Signal. 13, 269–278. 10.1089/ars.2009.2952 20059401

[B107] Viña J. Gambini J. García-García F. J. Rodriguez-Mañas L. Borrás C. (2013). Role of oestrogens on oxidative stress and inflammation in ageing. Horm. Mol. Biol. Clin. Investig. 16, 65–72. 10.1515/hmbci-2013-0039 25436748

[B108] Wanagat J. Dai D. F. Rabinovitch P. (2010). Mitochondrial oxidative stress and mammalian healthspan. Mech. Ageing Dev. 131, 527–535. 10.1016/j.mad.2010.06.002 20566356 PMC2933331

[B109] Webster M. J. Herman M. M. Kleinman J. E. Shannon Weickert C. (2006). BDNF and trkB mRNA expression in the hippocampus and temporal cortex during the human lifespan. Gene. Expr. Patterns 6, 941–951. 10.1016/j.modgep.2006.03.009 16713371

[B110] Wei S. M. Baller E. B. Kohn P. D. Kippenhan J. S. Kolachana B. Soldin S. J.. (2018). Brain-derived neurotrophic factor Val^66^Met genotype and ovarian steroids interactively modulate working memory-related hippocampal function in women: a multimodal neuroimaging study. Mol. Psychiatry 23, 1066–1075. 10.1038/mp.2017.72 28416813 PMC10103851

[B111] Wu M. V. Manoli D. S. Fraser E. J. Coats J. K. Tollkuhn J. Honda S.. (2009). Estrogen masculinizes neural pathways and sex-specific behaviors. Cell 139, 61–72. 10.1016/j.cell.2009.07.036 19804754 PMC2851224

[B112] Yang S. H. Liu R. Perez E. J. Wen Y. Stevens S. M. Jr. Valencia T.. (2004). Mitochondrial localization of estrogen receptor β. Proc. Natl. Acad. Sci. U S A 101, 4130–4135. 10.1073/pnas.0306948101 15024130 PMC384706

[B113] Yao J. Hamilton R. T. Cadenas E. Brinton R. D. (2010). Decline in mitochondrial bioenergetics and shift to ketogenic profile in brain during reproductive senescence. Biochim. Biophys. Acta 1800, 1121–1126. 10.1016/j.bbagen.2010.06.002 20538040 PMC3200365

[B114] Yin F. Boveris A. Cadenas E. (2014). Mitochondrial energy metabolism and redox signaling in brain aging and neurodegeneration. Antioxid. Redox Signal. 20, 353–371. 10.1089/ars.2012.4774 22793257 PMC3887431

[B115] Yin F. Yao J. Sancheti H. Feng T. Melcangi R. C. Morgan T. E.. (2015). The perimenopausal aging transition in the female rat brain: decline in bioenergetic systems and synaptic plasticity. Neurobiol. Aging 36, 2282–2295. 10.1016/j.neurobiolaging.2015.03.013 25921624 PMC4416218

[B116] Yu Z. Sunchu B. Fok W. C. Alshaikh N. Pérez V. I. (2015). Gene expression in the liver of female, but not male mice treated with rapamycin resembles changes observed under dietary restriction. Springerplus 4:174. 10.1186/s40064-015-0909-7 26034704 PMC4447730

[B117] Yurek D. M. Fletcher-Turner A. (2001). Differential expression of GDNF, BDNF, and NT-3 in the aging nigrostriatal system following a neurotoxic lesion. Brain Res. 891, 228–235. 10.1016/s0006-8993(00)03217-0 11164827

[B118] Zárate S. Stevnsner T. Gredilla R. (2017). Role of estrogen and other sex hormones in brain aging. Neuroprotection and DNA repair. Front. Aging Neurosci. 9:430. 10.3389/fnagi.2017.00430 29311911 PMC5743731

[B119] Ziegenhorn A. A. Schulte-Herbrüggen O. Danker-Hopfe H. Malbranc M. Hartung H. D. Anders D.. (2007). Serum neurotrophins—a study on the time course and influencing factors in a large old age sample. Neurobiol. Aging 28, 1436–1445. 10.1016/j.neurobiolaging.2006.06.011 16879899

